# Dance program for physical rehabilitation and participation in children with cerebral palsy

**DOI:** 10.1080/17533015.2011.564193

**Published:** 2011-06-13

**Authors:** Citlali López-Ortiz, Kim Gladden, Laura Deon, Jennifer Schmidt, Gay Girolami, Deborah Gaebler-Spira

**Affiliations:** ^a^ Rehabilitation Institute of Chicago, Chicago, IL, USA; ^b^ Department of Physical Medicine and Rehabilitation, Feinberg School of Medicine, Northwestern University, Chicago, IL, USA; ^c^ Pathways Center, Glenview, IL, USA

**Keywords:** dance, physical rehabilitation, participation, cerebral palsy, children

## Abstract

*Objective*: This pilot study aimed to examine a classical ballet program created for children with cerebral palsy (CP) as an emerging physical rehabilitation modality. The main program goals were to promote participation and to provide an artistic, physically therapeutic activity. *Methods*: The study was conducted in collaboration with a tertiary rehabilitation hospital, one outpatient physical therapy clinic, and one community center. As a pilot exploratory study, the research design included questionnaires to assess the participants' (children (*n* = 16), parents (*n* = 16), and therapists (*n* = 13)) perceptions on the therapeutic benefit of the dance program. A binomial statistical model was adopted for the analysis of the results. *Results*: Main results were that the children reported high enjoyment level (*p* < .0001) and desire for more classes (.0001); the parents reported perceived therapeutic benefit (*p* < .0001); and the therapists viewed the class as a positive adjunct to therapy (*p* < .0001). *Conclusions*: The main limitation of this work was the utilization of subjective outcome measures. However, this is the first step toward the development of objective measures of an intervention that, to our knowledge, has not been analyzed in the past. We conclude that the program has the potential of developing into an evidence based rehabilitation resource for children with CP.

## Background

The prevalence of cerebral palsy (CP) in children is estimated to be, on average, between 1.5 and 2.5 cases per 1000 live births (Paneth, Hong & Korzeniewski, 2006). Children with CP face challenges in movement and posture control. Hypertonia and various combinations of motor impairments such as weakness, reduced selective motor control, poor balance and discoordination are common in children with CP. These deficits contribute to impaired movement and posture that compromise adequate function and participation in social activities (van der Heide et al., 2004; Woollacott et al., 2005). Thus, techniques to enhance functional mobility are critical to improving outcomes in pediatric CP. Movement ability impacts learning through the child's exploration and manipulation of the environment. In addition, movement is the basis for many human experiences, such as community activities, play and cognitive development. The International Classification of Functions (ICF) of the World Health Organization (WHO) provides a universal starting point for a comprehensive understanding of the human experiences of functioning and disability where the physical, social and environmental factors become intertwined (Cieza & Stucki, 2008). According to the ICF, participation consists of taking part or being involved in everyday life activities and roles. Thus, participation in leisure activities has emerged as an important health outcome for children with disabilities. Leisure activities are typically those in which an individual freely chooses to participate during free time because it is enjoyable. Benefits of participation in leisure activities include fostering friendships and developing personal interests and identity (Majnemer et al., 2008). In children with CP, impaired mobility leads to decreased participation in the community and reduced contact with peers in activities and play (Fauconnier et al., 2009; Imms, 2008; Michelsen et al., 2009; Parkes, McCullough & Madden, 2010; Pratt, Baker & Gaebler-Spira, 2008; Shikako-Thomas, Majnemer, Law & Lach, 2008). As a consequence, the development of cognitive, motor and social skills is compromised (Bottcher, 2010; King et al., 2009). This decrease in participation correlates positively with increased gross motor impairments (Parkes et al., 2010). Furthermore, it has been hypothesized that attentional and executive deficits present in children with CP may also contribute to the decrease in societal participation (Bottcher, Flachs & Uldall, 2010). The net effect is a negative feedback loop on the development of the child as a whole. Indeed, participation intensity of children with CP in recreational, active physical and social activities tends to decrease through time (King et al., 2009). It has been noted, however, that participation in self-improvement activities and skill-based activities such as dance does not seem to decline through time (King et al., 2009). The need for activity-based therapies in CP has been stressed in the recent literature (Damiano, 2006), since children with CP have restricted access to skill-based movement programs that combine therapeutic principles of movement rehabilitation with community involvement. We created and established a dance program based in classical ballet technique for children with CP to enhance their movement abilities, artistic expression, socialization and participation. The main objective of the dance program was to deliver physical rehabilitation through the execution of artistic movement in a group setting where the children participated by their own choice and perceived enjoyment. We considered the child's right to choose to participate essential, as it has been reported that motivation seems to be key for successful treatment in children with CP (Kwak, 2007; Majnemer et al., 2008; Morris, 2009). Additionally, by allowing the children to participate of their own choice we enhanced the leisure component of the dance class while involving the children in therapeutic movement activity.

Assisting the motor and cognitive development of the child while preventing secondary injury is the main goal of treatment in CP (Sanger, 2008). Physical and occupational therapy are standard treatment and the mainstay management for the motor impairments. Although further research is needed to establish the effectiveness of various forms of therapy, rehabilitation commonly focuses on a range of motion exercises to prevent and delay contractures (continued contraction of a muscle in the absence of stimuli), flexibility exercises to increase range of motion, progressive resistance exercises to improve strength and various interventions to improve posture, balance and the acquisition of functional skills (Anttila, Suoranta, Malmivaara, Makela & Autti-Ramo, 2008a; Anttila, Autti-Ramo, Suoranta, Makela & Malmivaara, 2008b; Damiano, 2009). The intensity and frequency of therapy vary across children, but many have weekly therapy that extends into adulthood. Most therapy is delivered on an individual basis. Therapy may take the place of time that would have been devoted to group activities typical of normally developing children. Current literature stresses the need for newer, task-related and intense programs of therapy with life-style modifications (Bower, Michell, Burnett, Campbell & McLellan, 2001; Damiano, 2006, 2009). Given the marginalization that many children with CP experience, dance can be valuable both physically and socially, as well as being an introduction to an art form that is both aesthetic and athletic. Thus, therapeutic movement that enhances motor control and mimics community activity answers several concerns for rehabilitation of children with CP. Our pilot class was based on these concerns and focused on two domains of the ICF namely: Body Structures and Functions, and Activities and Participation. We report on the rationale, implementation and design of the dance program for children with CP and on a pilot study intended to survey the therapeutic benefits and modifications in participation related to the dance class as perceived by the children, parents and therapists involved (Lopez-Ortiz, Gladden, et al., 2009).

The paradigm of dance, and in particular of classical ballet technique, offers a well-tested language-like representation of whole-body movements as sequences of constitutive elements endowed with static and dynamic stability that is suitable for quantitative analysis. The inherent static and dynamic stability of classical ballet vocabulary is largely due to the use of positions and movements that are guided by the limits of the mechanical range of motion of the joints and/or by actively stabilizing the joints in such positions. Since canonical positions and movements are defined within the anatomical Cartesian planes, quantitative analysis is simplified. Moreover, classical ballet technique organizes body movement in space and time with a language structure where static positions become the building blocks or primitives for the creation and organization of complex and rich motor performance. Elite dance training has modular and hierarchical organization similar to some physical therapy techniques. Conservatory ballet training involves the training of static postures connected by, initially, slow movements (Kostrovitskaya, 2004). As training advances, the movements are executed at greater speeds and the static postures may blend into the movement. Therefore, in early training balletic movement tasks are in fact a sequence of point-to-point reaching movements in free space. Point-to-point reaching movements have been successfully studied and used for stroke recovery (Rohrer et al., 2004). Movement control in stroke survivors presents similar characteristics to that of children with CP, such as spasticity, discoordination and reduced selective muscle activation patterns. Thus, the inclusion of the ballet arm postures and movement for training is consistent with current perspectives on motor control and motor learning by “compositionality” (Hammer, 2003; Krebs, Aisen, Volpe & Hogan, 1999; Miyamoto, Morimoto, Doya & Kawato, 2004; Morasso & Mussa Ivaldi, 1982). Here, compositionality refers to the construction of movement trajectories by combination of building blocks or primitives. The movement primitives in this consist of movement trajectory segments that that concatenate in the points of minimum velocity.

**Figure 1:**
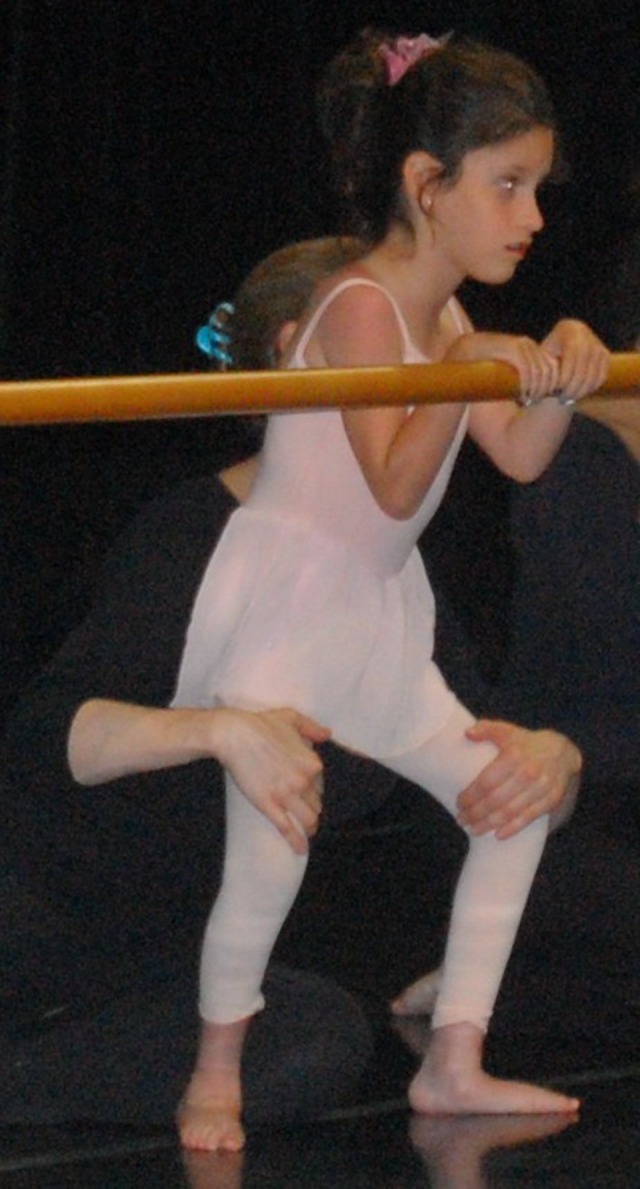
Second Position of the Feet with *Demi-plié* (knee flexion). Child with GMFCS level IV. Care is taken to encourage proper musculoskeletal alignment using the *barre* for trunk support through the upper limbs while executing *demi-plié*. Photo reproduced with permission.

As an art form, classical ballet technique and training principles systematically enhance alignment, flexibility, core strength, postural control and selective motor control. All these training goals are shared in the rehabilitation of children with CP. It is known that children with CP have less joint position sense as compared to neurologically intact children, with bias in the direction of internal rotation of the hip and pronation of the forearm (Wingert, Burton, Sincalir, Brunstrom & Damiano, 2009). One example of a ballet movement that addresses this bias is the practice of external rotation and abduction of the hip joints that leads to a stance with the heels separated in the coronal plane known as *second position* in classical ballet terminology (see Figure 1). In this pilot dance program, augmentation of sensory information by tactile cues and close volunteer work aid in accommodating for the lack of sensory input. Positions that counter possible orthopedic deformity due to CP were practiced while avoiding physical pain. For the children with intellectual impairments, the ability to follow three-step directions was a prerequisite for enrollment. We assumed that children with borderline IQs could learn the basic routine of dance exercises, as well as execute the sequence of movements that were part of each class. Medical co-morbidities influence the child's experience of the dance class. Sensory deficits such as hearing occur in up to 30% of children with CP and vision disturbances may be seen in up to 70% of children with CP (Ghasia, Brunstrom, Gordon & Tychsen, 2008; Venkateswaran & Shevell, 2008). These deficits limit processing of information and reduce visual and motor learning (Ostensjo, Carlberg & Vollestad, 2003). Seizures, intellectual disability and medications could reduce the attention to task and learning (Himmelmann, Beckung, Hagberg & Uvebrant, 2006). Since dance has already been proven effective in improving motor and mental scores of patients with Parkinson's disease (Erhardt, 2009), we expected positive outcomes in the children enrolled in the ballet-based dance class.

Dance classes include music for the execution of movement. It has recently been suggested that early experiences during development encourage the simultaneous and interconnected wiring of movement and auditory representations in the brain (Trainor, 2008). There is recent evidence of the presence of auditory mirror neurons that seem to be involved in tracking rhythmic auditory events in anticipation of their use in conjunction with the motor system (Chen, Penhune & Zatorre, 2008). Thus, it was plausible to expect that the execution of movement would be facilitated by the presence of a beat or musical rhythm in at least some forms of CP. A preliminary study in children with CP of Gross Motor Function Classification Score (GMFCS) III and IV showed that music induces movement trajectories of the arms in ballet-like point-to-point reaching movements that exhibit less abnormality of curvature and increased blending of movement segments as compared to the no-music condition (Lopez-Oritz, Gladden, et al., 2009; Lopez-Oritz, O'Shea, Mussa-Ivaldi & Gaebler-Spira, 2009). In light of this evidence, the dance program included live music.

## Methods

Two dance classes were created, one for children with GMFCS I and II and one for children with GMFCS III and IV. The children enrolled by their own choice with the support of their parents. The program was supported by a tertiary rehabilitation hospital, one outpatient physical therapy clinic and one community center for children. Volunteers from the medical community and some parents assisted during the class. The classes were held once weekly in session of five to eight weeks depending on location availability. The children were surveyed only once at the end of the first full session. Eight children, their assistants, teacher and pianist participated in a demonstration of the dance class in a formal dance recital. The children and their parents were surveyed again after the performance.

Classical ballet training is progressive and repetitive, thereby allowing the student to perform movements consistently for mastery and to achieve the smooth execution of complex movement phrases. Preparatory conditioning exercises were developed with progressive levels of difficulty, with the end goal of executing the following classical ballet exercises.

• At the ballet *barre*: balletic posture control (voluntary effort to achieve anatomically correct alignment of the skeleton), *demi-plié* (bending of the knees while keeping the heels in full contact with the floor), *relevé* (raising of the heels with straight knees until full ankle flexion is achieved), positions of the feet (first position: the heels are together with the lateral rotation of the femur leading to moderately turned out feet, second position: the feet are separated at approximately one foot distance, third position: the heel of one foot is pressed against the lateral aspect of the arch of the other foot, fourth position: one foot is placed in front on the other while still maintaining the outward rotation at the hips, fifth position, the heel of one foot is placed in front of the toes of the other foot), *battement tendú devant* and *à la seconde* (one leg reaches to the front or the side while maintaining the hip turnout on both legs and extended knees, the foot brushed the floor to reach full plantar flexion in all joints), *retiré* (abduction of the hip joint while the knee bends to allow the fully plantar flexed foot to slide on the supporting leg), *relevé lent devant* (slow hip flexion abduction and extension while maintaining turn out, extended knees and full planter flexion of the foot), *grand battement devant* (fast hip flexion motion with extended knees and fully plantar flexed feet), *demi-ramassé* (trunk flexion from the hip joint while maintaining a neutral position of the vertebral column), *cambré* (back extension while attempting to maintain the lower limbs in a vertical position) (Kostrovitskaya & Pisarev, 1995).
• In the center: balletic posture control, *plié*, *relevé*, positions of the feet, *battement tendú devant* and *à la seconde*, *retiré*, *relevé lent devant*, positions of the arms and port de bras, *sautés* (jumps starting and ending with *demi-plié*), gallops, balletic walking, balletic running (quick small steps in *relevé*). The preparatory exercises were typically performed prone or supine in a group circle formation. As strength and selective motor control improved, the exercises were executed with the ballet bar as support, in the center in a group circle formation, or along diagonals across the floor.

The children with GMFCS I and II were typically assisted by one or two volunteers while the children with GMFCS III and IV were typically assisted by two or three volunteers depending on their individual needs. The presence of a pianist, or pianist and two violin players in the class was instrumental to establish an adequate tempo, time signature and desired movement quality for the execution of the movements and postures. Table 1 shows the topics covered in the dance class that impact to body structures and functions according to the ICF framework. Similarly, Table 2 lists the aspects of activities and participation included in the design of the dance class within the framework of the ICF.

**Table:** Table 1 Body Structures and Functions Items of the ICF (WHO) included in Dance Training for Children with Cerebral Palsy.

**Table:** Table 2 Activities and Participation Items of the ICF (WHO) included in Dance Training for Children with Cerebral Palsy.

Surveys were developed based initially on the LIFE-H questionnaire (Noreau et al., 2007) and questions were created to investigate specific aspects germane to the dance class not reflected in the pertinent Life-H questions. The surveys focused on participation and perceived therapeutic benefit from the dance class and dance performance. We developed questionnaires specific for the parents, children and the therapists involved in the program. The questions are presented along with the results in Tables 3–5. We obtained informed consent/assent from all the participants before completion of the surveys as approved by the Institutional Review Board of Northwestern University.

The results of the questionnaires that corresponded to a yes or no answer were converted to a numerical scale with YES = 1 and NO = –1. A binomial statistical test was performed on the numeric results to establish the evidence against the null hypothesis that a negative or positive response had equal random probability of occurrence (Snedecor & Cochran, 1996). In other words, a small *p*-value indicated a strong positive trend in the data. Additional comments reported in the survey that could not be scored in a numeric scale are included in Tables 3–5.

## Results

The results are presented in Table 3 for the children's survey, Table 4 for the parents’ survey and Table 5 for the therapists’ survey. The children expressed the desire for more classes (*p* < .0001), a high enjoyment level (*p* < .0001), new interest in participation in a school group (*p* < .04), new interest in watching a dance show (*p* < .0001) and new interest in attending an art show (*p* < .004). The parents rated the class highly with overall enjoyment (*p* < .0001), therapeutic benefit (*p* < .0001), positive influence in other ongoing therapy (*p* < .04) and all would enroll their children again. Parents’ comments included “I notice improved behavior, happier, enjoying the activities”, “They loved it, and they don't feel like they are in a therapy room”. The therapists identified advantages working in the dance class setting (*p* < .002), would make changes in their personal therapy sessions because of this program ( *p* < .002), had new treatment ideas as a result of their participation in the class ( *p* < .04), felt that the children gained benefits that might not be achieved in a typical therapy program ( *p* < .01) and viewed the class as a positive adjunct to traditional therapy ( *p* < .0001). The parents did not perceive any improvements in head, trunk, arm and leg control. Therapists’ comments included “This setting is very conductive for socialization and hands-on approach”, “I am always looking for participation-based treatment ideas and dance incorporates so many rehabilitation principles and [this is] more motivating and engaging [than other forms of therapy]”, “A chance to be with a group of friends with common disabilities is always an encouragement”, “allows children to explore and integrate other aspects of self”. One therapist commented on the possibility of improving the dance class by incorporating quantitative progression measures every class, while another desired incorporation of specific functional goals. Overall, the main areas of improvement suggested were location, accessible parking, time of class, musical variety and qualitative outcome measures.

**Table:** Table 3 Results of the Children Survey. The “Comments” column includes the general trends in the responses. For the question, *Did you like the class?* the children answered to: *Please color in the face below that best shows how much you liked or didn't like the class:* 
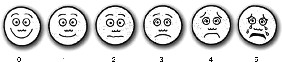
For the statistical purposes, all the answers of levels 0 and 1 were pooled as “yes” levels 2 to 5 were polled as “no”.

Children (*n* = 16)			
Questions	Yes	*p*-value	Comments
Would you like to take more dance classes?	15	< .0001*	
Was this fun?	16	< .0001*	
Are you interested in joining a school group?	11	< .04*	
Are you interested in joining a group in your town?	8	< .4	
Are you interested in watching a dance show?	13	< .0001*	
Are you interested in going to see some other kind of art show?	11	< .04*	
Did you like the class? (see details in legend)	16	< .0001*	
What did you enjoy the most about the class?			Environment, bars, mirrors, dance, music
What would make it more fun?			Friends from school, Music variety, Focus on port de bras, Increase in class size
Did you learn something new?			Specific dance movements, Ballet is fun, Music and dance with piano, How to dance
*denotes statistical significance			

**Table:** Table 4 Results of the Parent Survey. Sample comments are quoted in the last column. Comments without quotes reflect the general trend in the responses.

Parents (*n* = 16)			
Questions	Yes	*p*-value	Comments
What are you overall impressions of the dance experience?	15	< .0001*	Overall enjoyment Artistry Relaxation “I think it is great to have. It gave my son something normal to do” “nice to have a class for special needs to participate in without feeling self conscious”
Was the class enjoyable?	14	< .001*	“It is a lot of fun and very enjoyable”
Do you think the class provided therapeutic benefit?	14	< .001*	“The graceful movements were therapeutic in a way that made the girls aware of a different feel”
Did you notice any changes in your child's performance in their regular therapy sessions while involved in this dance program? If yes, what changes did you observe?	11	< .04*	“developed more confidence” “movements are more controlled and precise”
Did you notice improvement in your child's head control as a result of this dance program?	9	< .22	
Did you notice improvement in your child's trunk control as a result of this dance program?	8	< .4	
Did you notice that your child had improved control of his/her arms or legs as a result of this dance program?	5	< .9	
Was anything stressful for you or your child?	6	< .8	“long commute, hard at first but got used to it” “yes, she is too young for the class” “driving in the snow” “earlier time slot would be better”
What differences do you see between dance and therapy?	12	< .01*	“Important that she did this with her peers” “the group dynamic was excellent” “any kind of creative expression – music, art, dance, can be extremely therapeutic” “she doesn't see dance class as work” “dance is more for enjoyment” “dance is more enjoyable for her” “dance is more fun and less stress”
Would you recommend this class to others?	16	< .0001*	“Absolutely”
Would you enroll your child again?	15	< .0001*	
What could improve the experience?			More classes (*n* = 3) Satisfied (*n* = 4) “Classes closer to home” “Teacher and assistants are wonderful”

**Table:** Table 5 Results of the Therapists’ Survey. Sample comments are quoted in the last column. Comments without quotes reflect the general trend in the responses.

Therapists (*n* = 13)			
Questions	Yes	*p*-value	Comments
What are the advantages that you see working in this setting?	11	< .002*	Teacher student ratio
			Environment
			Creativity
			Socialization
			Novel therapy
			“Advantages with coordination muscle tone, self esteem, social development”
What are the disadvantages that you see working in this setting?	4	< .9	Location
			Lack of quantitative progression measures in class
			“I would like functional goals”
How could the class be improved to provide more benefit that is therapeutic?	5	< .7	Home program exercise
			Slow down program
Will you change your practice as a result of your participation in this class?	11	< .002*	New kinesthetic queuing
			Participation-based treatment ideas
			Creative ideas and types of symbolism
			Development of empathy and patience with patients
Do have any new treatment ideas as a result of participating in this program?	11	< .04*	“Incorporating breathing with movement”
			“Arm movement”
			“Rhythm coupled with movement”
			‘More relaxation techniques”
			“Music to home exercise program”
			“A whole class on just port de bras”
			“Body-based practice”
			“Tactile guidance for alignment”
What benefits do you feel the children gained from this	10	< .01*	“Movement to music that was appropriately paced”
program that might not be achieved in a typical therapy program?			“Transitions are smoother in dance class”
			“Socialization”
			“Alternative structure from traditional therapy”
			“Body as a creative soul”
			“Self confidence”
			“Exposure to music and rhythm”
			“Motivation and excitement”
What is your overall impression of the program as an adjunct to traditional therapy?	13	< .0001*	“All the families enjoyed having an activity on Saturdays”
			“Dance is a nice way to capture more holistic view of child-physical movements with emotions”
			“Offers the children something fun that doesn't feel like work”
			“Integration of other aspects of self”
			“It's a wonderful concept especially for young children”
What were the dissimilarities to a therapy session?			Class structure
			Peer contact
			Less hands on
			Less “breaks” between activities
*denotes statistical significance.			

Eight children participated in a formal dance performance at a higher-level educational institution. The children were excited and cooperative. Although one child showed signs of stress before the performance, she expressed pride in her achievement once the performance had ended. As a group, the children that participated in the performance expressed enjoyment in: dancing, dancing with their friends, being watched by other people, being on stage and receiving a standing applause from the audience. All the children surveyed in this group (*n* = 6) reported having fun during the performance and expressed interest in participating in more performances. The children who participated in the dance performance showed improved attendance to the classes after the performance and look forward to having more classes.

## Discussion

A classical ballet based dance class provides an organized systematic approach to movement dexterity development that has been created and perfected through centuries of practical experience. Several of its training principles are aligned with physical therapy practices, but in addition it provides a language structure to movement that is absent in other forms of physical training. This language structure allows for the coordination of complex movements far beyond those required for daily life. Within its intrinsic organization, classical ballet positions and steps act to counter the main movement deficits present in CP from flexibility, to postural control, to selective motor control. This places ballet training as a key instrument for the rehabilitation of children with CP. Moreover, since ballet training is an expressive art, it incorporates cognitive, emotional and behavioral functions that are not necessarily part of traditional therapy. When presented as an optional movement activity for the children's enjoyment, they do not perceive it as “work” or “therapy”. The inclusion in each class of children of similar ability provides a less stressful environment in which they can relate with their peers and happily encourage each other during the class. Individualized attention from the assistant therapists to each child and live music created a group class environment in which somatic empathy (i.e. attention to non-verbal, somatic cues, such as posture and gesture thereby gaining insight into what the child may be experiencing) from the instructor and therapists allowed each child to learn new movements and enjoy the process. This somatic empathy may be key for improved physical rehabilitation outcomes in cerebral palsy rehabilitation (Chaitow et al., 2010). Empathy was also present in the musicians that played the music for the class and accommodated the mood changes and movements qualities required in each exercise.

Future plans include addressing the research and location shortcomings as well as increasing the enrollment and frequency of the dance classes. We envision creating dance pieces for performances in addition to formal class demonstrations. In these performances, as in the dance class demonstrations, emphasis will be placed in the movements and dancing of the children, while the assistants strive to remain in the background provided support as needed.

Challenges to be addressed include the quantitative evaluation of the physical mobility outcomes, a validated questionnaire specific to the dance class and location accessibility.

Quantitative evaluation of movement characteristics remains statistically elusive as class sizes are small and variability of conditions and age is large. Additionally, the qualitative nature of the outcome measures is a weakness of the present study. However, as a pilot exploratory study, we observed potential benefits in this intervention and the next step will be to incorporate validated quantitative assessments of progress. Despite the need for improvement in these areas, given that the children enrolled in the ballet class by their own choice and with parental support and they all reported enjoying the class, at the very least, they will have a positive memory of a creative activity in which their own body and movements are the tools of expression.

## Conclusions

The surveys revealed that this program incorporates key parental and ICF goals in occupational and physical therapy, such as the importance of facilitating improved motor control, enhancing posture and trunk stability and coordinating motor movements in response to verbal commands and visual cues. From a therapy perspective, when such goals are addressed in an environment that is engaging for the child, motivation and participation increase. The group setting creates natural peer modeling and promotes social interaction that is incredibly valuable to children who otherwise spend much of their therapeutic time in one-on-one settings with adults. This class improves the child's repertoire of activities and appreciation of a skilled art. The dance program allowed children to develop interest and appreciation of an art form that has the potential to enhance their lives, physically, creatively and emotionally. The enjoyment of the class by all the persons involved provided a positive therapeutic environment for the children and their families. There is a need for better outcome measurement tools in the qualitative and quantitative aspects on the class. Combining movement and expressive art in the dance class, augments the potential for improving children's posture, movement abilities and societal participation as well as generating rewarding experiences throughout a lifetime.
